# Inhibition of Zymosan-Induced Inflammatory Factors Expression by ATRA Nanostructured Lipid Carriers

**DOI:** 10.1155/2016/4952340

**Published:** 2016-06-01

**Authors:** Hongyan Zhou, Wensong Zhang, Xunyi Gao, Hongguang Zhang, Ning Kong

**Affiliations:** ^1^Department of Ophthalmology, China-Japan Union Hospital of Jilin University, Changchun 130033, China; ^2^Department of Ophthalmology, The Second Hospital of Jilin University, Changchun 130000, China; ^3^Department of Chemical Engineering and Application, College of Chemistry, Jilin University, Changchun 130000, China; ^4^Department of Pharmacy, College of Pharmacy, Jilin University, Changchun 130041, China

## Abstract

*Purpose*. The study aimed to evaluate the effect of all-trans retinoic acid-loaded nanostructured lipid carriers (ATRA-NLCs) on the zymosan-induced expression of the cytokines IL-4, IL-10, and IFN-*γ* and the matrix metalloproteinases/tissue inhibitor of metalloproteinases (MMPs/TIMPs) and TLR_2_ in rabbit corneal fibroblasts (RCFs).* Methods*. ATRA-NLCs were prepared by emulsification. RCFs were isolated and harvested after four to seven passages in monolayer culture. Cytokine release (IL-4, IL-10, and IFN-*γ*) induced by zymosan was analyzed by cytokine release assay, reverse transcription, and real-time polymerase chain reaction (RT-PCR) analysis detection. MMP-1, MMP-3, and MMP-13, TIMP-1 and TIMP-2, and TLR2 expression were analyzed by immunoblotting.* Results*. ATRA-NLCs were resistant to light and physically stable, and the average size of the ATRA-NLCs was 200 nm. ATRA-NLCs increased the zymosan-induced release of IL-4 and IL-10 and decreased the release of IFN-*γ* by RCFs. ATRA-NLCs decreased the levels of TLR2 and MMPs/TIMPs above.* Conclusions*. ATRA may be a potent anti-inflammatory agent for the therapy of fungal keratitis (FK).

## 1. Introduction

Fungal keratitis (FK) is an ulcerative and sight-threatening infection of the cornea that sometimes leads to blindness. Although patients with FK have been treated with antifungal drugs that prevent the progression of corneal pathogenesis, further study is still needed. The objectives of this study were to investigate the effect of ATRA-NLC on the zymosan-induced expression of the cytokines IL-4, IL-10, IFN-*γ*, MMPs/TIMPs, and TLR_2_.

Zymosan, a *β*-glucan component of the yeast cell wall, is a ligand of TLR_2_ and dectin-1 which stimulates macrophages to produce proinflammatory mediators [1]. Zymosan can also regulate TLR_2_ gene expression in the context of neuroinflammation [2]. Fungal inflammation is mediated by the expression of TLR_2_ activation. TLRs may activate the innate immune system. TLRs have been used as a legitimate therapeutic strategy. Zymosan-induced TLR_2_ expression was investigated in this study.

MMPs are enzymes involved in leukocyte diapedesis and migration to the inflammatory focus. MMP-2, MMP-9, and TIMP-1 play specific roles in the pathogenesis of FK [3]. Specifically, the levels of MMP-8, MMP-9, MMP-13, and TIMP-1 increase during the early stages of* C. albicans* keratitis [4], and the ratio of MMPs/TIMPs is higher in the FK corneas. Corneal polymorphonuclear neutrophils (PMNs) that infiltrate the cornea of patients with FK increase the activities of MMP-8 and MMP-9, thereby enhancing tissue destruction [5]. Therefore, this study was to determine the association between the MMP/TIMP and zymosan-induced inflammation to clarify the role of MMP/TIMP in the pathology of FK and then to study the potential role of new therapeutic method.

IL-4 and IL-10 play a role in protective immunity against infections with extracellular parasites and are responsible for inflammatory diseases. IFN-*γ* is secreted by activated Th1 and other immune cells, and it creates proinflammatory microenvironments [6]. Zymosan-induced production of the cytokines IL-4, IL-10, and IFN-*γ* by RCFs was examined in this study.

Nuclear receptors are ligand-activated transcription factors that signal the expression of the genome. Understanding the expression of receptors and their activating lipid ligands in immune diseases will be crucial for the development of new therapies to target nuclear receptors [7]. ATRA is a bioactive derivative of vitamin A that has demonstrated immunomodulatory effects in many immune disorders [8]. ATRA is a crucial immunostimulatory cofactor that activates macrophages and their subsequent differentiation into dendritic-like cells [9]. Moreover, ATRA exerts an anti-inflammatory effect on monocytes via TLR_2/1_ and CD14 expression [10, 11]. ATRA exacerbates allergic immune and inflammatory responses, most likely by promoting Th2 development [12]. Thus, ATRA may be a novel strategy to treat inflammation in humans, but it is poorly soluble, which hinders its ocular delivery. In this study, we designed NLCs to overcome these barriers, which led to poor bioavailability. Because ATRA exhibits anti-inflammatory activity [10], we investigated the effect of ATRA-NLCs on the zymosan-induced expression of TLR_2_, IL-4, IL-10, and IFN-*γ*, as well as the function of MMP/TIMP in RCFs. The potential role of ATRA-NLCs in the therapy of FK was clarified.

## 2. Materials and Methods

 Dispersant, collagenase, Eagle's minimum essential medium (MEM), Dulbecco's phosphate-buffered saline (DPBS), Fetal Bovine Serum (FBS), (Sigma), ATRA (Sigma Aldrich), Whales wax palm, soybean oil (Aladdin Reagent company), lecithin (Shanghai Huishi Biochemicals company), dibutylhydroxytoluene (chemically pure, Sinopharm Group Chemical Reagent company), oleic acid (chemically pure, Recovery of Tianjin Institute of Fine Chemicals), and Poly-Yamanashi Ester, 80 (East China reagent factory in Tianjin) were used. The following antibodies were used: MMP-1 and MMP-3 and TIMP-1 (RD), MMP-13 (BioVision), TIMP-2 and TLR_2_ (Boster), and GAPDH (Cell signal).

Anti-mouse IgG-HRP (Abcam) and goat anti-rabbit IgG-HRP (RD) were used. Trizol Reagent (Invitrogen) LightCycler® 480 Real-Time PCR System (Roche Diagnostics GmbH) and Primer were synthesized by Sangon Biotech Co. Ltd. (Shanghai, China). Reverse transcription system (Promega) was used. The following equipment was used: a DF-101S hot-type constant temperature heating magnetic stirrer (Shanghai Yukang Science and Education Equipment Co.), an Ultrasonic Cell Crusher (Ningbo Xingzhi Biotechnology Co.), a high-performance liquid chromatograph (Agilent), a ZF-I ultraviolet analyzer (Shanghai Gucun Electro-Optical Instrument company), a particle size analyzer (Brookhaven Instruments), and an electronic universal oven (Beijing everlasting light medical instrument company).

### 2.1. ATRA-NLC Preparation

**Figure 1:**
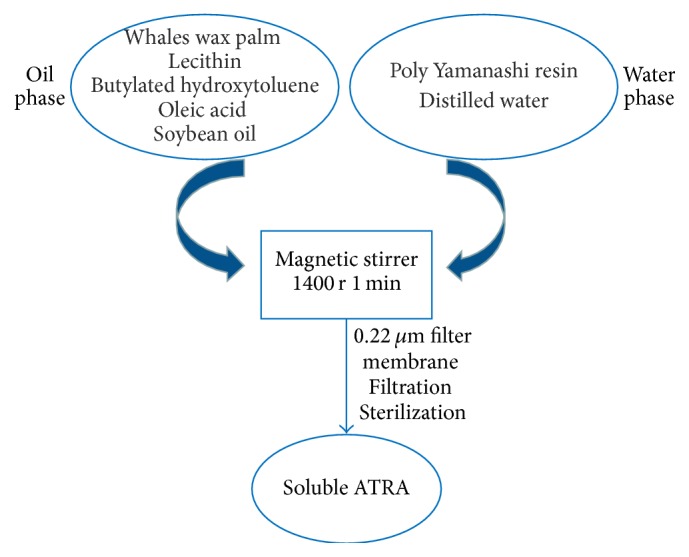
The method used to prepare ATRA-NLCs.

ATRA-NLC was prepared by emulsification according to the process shown in Figure 1.

### 2.2. Cell Isolation

New England white rabbits (2.0 to 2.5 kg) were obtained from the animal department of Jilin University. This study adhered to the ARVO Statement for the Use of Animals in Ophthalmic and Vision Research and was approved by the Animal Experimental Committee of Jilin University. RCFs were isolated as follows: briefly, the enucleated eye was washed with DPBS containing an antibiotic-antimycotic mixture; the endothelial layers and the epithelial sheets of the excised corneas were removed, and the remaining corneal tissue was cultured with collagenase (2 mg/mL, in MEM) at 37°C. Cells were harvested after four to seven passages in monolayer culture.

### 2.3. Cytokine Release Assay

RCFs (30000 cells/mL) were cultured in 24-well plates for 24 h with MEM alone. Serum-free RCFs were incubated with or without ATRA-NLC in the presence or absence of zymosan for 24 h. The IL-4, IL-10, and IFN-*γ* concentrations were then examined with a cytokine assay. Standard proteins were dissolved in MEM to generate standard curves. Data are presented as the mean values ± SD. ^*∗*^ *P* < 0.01 versus the corresponding value for cells cultured with zymosan (Dunnett T3). Each cytokine assay was repeated at least three times.

### 2.4. Quantitative Real-Time Polymerase Chain Reaction (RT-PCR) Analysis

RCFs were cultured in 60 mm dishes for 24 h with MEM alone. Serum-free RCFs were incubated with ATRA-NLCs for 1 hour and then for an additional 4 hours in the presence or absence of Zymosan. Total RNA was isolated from RCFs in 60 mm culture dishes with the use of Trizol Reagent and was subjected to RT. The resulting cDNA was subjected to real-time polymerase chain reaction (PCR) analysis with specific primers for IL-4, IL-10, and IFN-*γ* or glyceraldehyde-3-phosphate dehydrogenase (GAPDH) by the use of a LightCycler 480 Real-Time PCR System. The sequences of the PCR primers were designed by the literature previously [13]: the primers for IL-4 were GTTTCCCTGCTTTGAGATGG (forward) and TCAGGAAACAGCTTCGGAGT (reverse), the primers for IL-10 were TTTAGGCGAGAAGCTGAAGG (forward) and TCTTCACAGGGCAGGAATCT (reverse), the primers for IFN-*γ* were TGAACATGATGGATCGTTGG (forward) and CATTCACTTTGCTGGCAGTG (reverse), and the primers for GAPDH were AACTTTGGCATTGTGGAAGGA (forward) and AACATCATCCCTGCTTCCAC (reverse).

### 2.5. Immunoblot Analysis

Immunoblot analyses of MMP-1, MMP-3, MMP-13, TIMP-1, TIMP-2, and TLR_2_ were also performed as described previously [14]. In brief, cells (5 × 10^5^ cells per well of a 24-well plate) were cultured for 24 h in MEM and then incubated for 12 h with or without 0.01–0.1 *μ*mol/L ATRA-NLCs, followed by 30 min with or without zymosan (500 *μ*g/mL). The cell lysates (30 *μ*g of protein) were collected, and then the cell lysates were subjected to immunoblot analyses. Data are representative of three experiments.

## 3. Results and Discussion

### 3.1. ATRA-NLC Evaluation

ATRA-NLCs were light-resistant and physically stable, and they exhibited an average size of 200 nm. Precipitation and crystallization were not observed when the ATRA-NLCs were stored for one month at 4°C. High-speed centrifugal for 2 hours also works well.

### 3.2. Effects of ATRA-NLC on Zymosan-Induced IL-4, IL-10, and IFN-*γ* from RCFs

**Figure 2:**
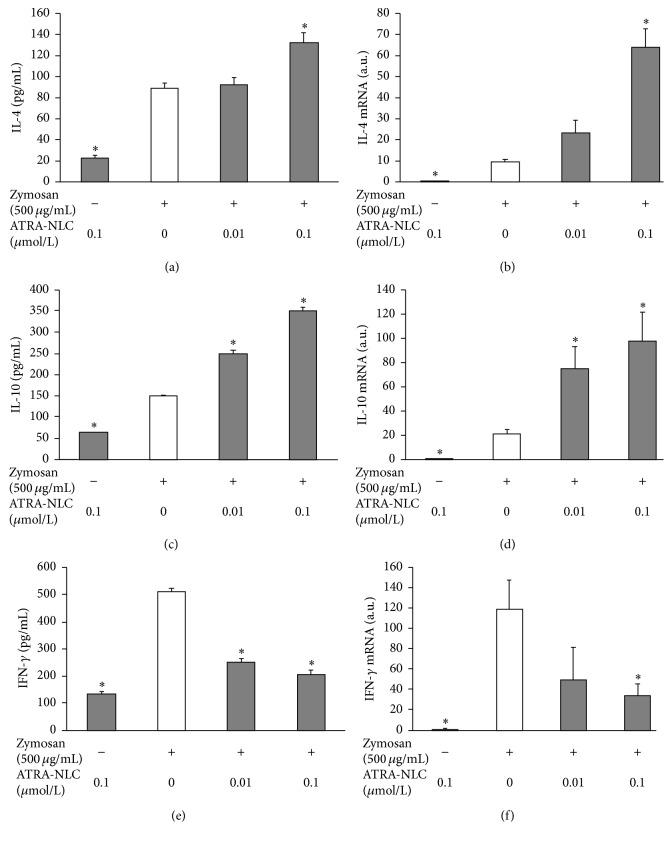
Effect of ATRA-NLC on the expression of IL-4, IL-10, and IFN-*γ* from RCFs and in RCFs. (a), (c), and (e) Serum-deprived RCFs were incubated in the absence or presence of ATRA-NLC with or without zymosan for 24 h. The IL-4, IL-10, and IFN-*γ* concentrations were examined at 24 h with a cytokine assay system. Zymosan can induce IL-4, IL-10, and IFN-*γ* release. ATRA-NLC further increased the release of IL-4 and IL-10 and decreased the release of IFN-*γ*. Data are the mean values ± SDs of three experiments. ^*∗*^ *P* < 0.01 versus the corresponding value for cells cultured with zymosan (Dunnett T3). (b), (d), and (f) RCFs were incubated in the absence or presence of the indicated concentrations of ATRA-NLC and zymosan, after which total RNA was isolated and subjected to RT and real-time PCR analysis of IL-4, IL-10, and IFN-*γ* mRNAs. Incubation of the cells with ATRA-NLC and zymosan induced the expression of IL-4 and IL-10 and decreased the expression of IFN-*γ* in a concentration-dependent manner. Data are the mean values ± SDs of three experiments. ^*∗*^ *P* < 0.05 versus the corresponding value for cells cultured with zymosan (Dunnett T3).

The effects of ATRA-NLC at various concentrations (0, 0.01, and 0.1 *μ*mol/mL) on the release of IL-4, IL-10, and IFN-*γ* induced by zymosan from RCFs were investigated first (Figures 2(a), 2(b), and 2(c)). Further, quantitative RT-PCR analysis revealed that ATRA-NLC increased the amounts of IL-4 and IL-10 mRNAs in RCFs induced by zymosan, and the amount of IFN-*γ* was inhibited in a dose-dependent manner (Figures 2(a), 2(b), 2(c), 2(d), 2(e), and 2(f)).

### 3.3. Effects of ATRA-NLC on MMP-1, MMP-3, and MMP-13 and TIMP-1 and TIMP-2 TLR_2_ Expression in RCFs

**Figure 3:**
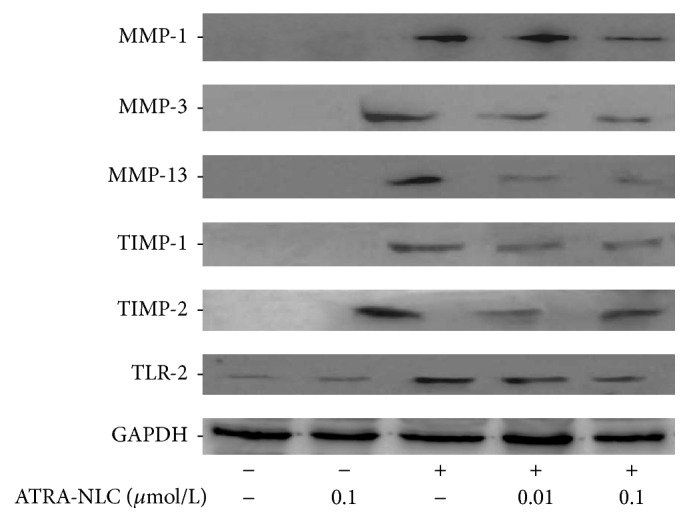
The effects of ATRA-NLCs on MMP-1, MMP-3, and MMP-13 and TIMP-1 and TIMP-2 TLR_2_ expression in RCFs stimulated with zymosan. RCFs were incubated with or without ATRA-NLC for 24 h and then treated with zymosan. The immunoblot analysis showed that ATRA-NLCs inhibited the zymosan-mediated stimulation of MMP-1, MMP-3, and MMP-13 and TIMP-1 and TIMP-2 TLR_2_.

RCFs were stimulated by zymosan. The RCFs were incubated with or without ATRA-NLCs (0.01–0.1 *μ*mol/L) for 24 h, followed by incubation with zymosan (500 *μ*g/mL) for 30 min. The immunoblot analysis showed that the levels of MMP-1, MMP-3, and MMP-13 and TIMP-1 and TIMP-2 TLR_2_ were increased by stimulation with zymosan, and this increase was inhibited by ATRA-NLCs (Figure 3).

## 4. Discussion

Natamycin 5% suspension, voriconazole, and topical amphotericin-B 0.015% constitute routine antifungal therapy for FK [15]. However, existing methods cannot meet the demands of clinical treatment, and new drugs are necessary. New antifungal therapies commonly target ergosterol, an essential constituent of fungal membranes that are not reluctant to microbial resistance and are minimally toxic to mammalian cells [16]. We have shown that ATRA-NLCs inhibited the zymosan-induced expression of the cytokines IFN-*γ*, MMPs/TIMPs, and TLR_2_ and increased the zymosan-induced expression of the cytokines IL-4 and IL-10 by RCFs.

The immune dominance of Th1 (IFN-*γ* and IL-2) may be characteristic of many immune-inflammation pathogeneses [17]. The Th1 response was maintained after infection because IFN-*γ* production is based mainly on CD4^+^ T cells [18]. Th1 cells are involved in all forms of dry eye, which is characterized by an inflammatory pathophysiology [19]. Th1 was expected to be a target in ocular anti-inflammatory therapy. Il-4 administration may inhibit the inflammatory response and the infiltration of phenotypic macrophages to suppress TNF-*α*-induced osteocyte apoptosis [12]. IL-4 and IL-10 can inhibit both Th1 cytokine expression and the consequent inflammation. Moreover, zymosan induces the expression of pro- and anti-inflammatory factors and Tregs. Specifically, zymosan increases the levels of IL-4, IL-10, and Foxp3-positive T cells [20]. ATRA can also inhibit the expression of Th1-associated (IFN-*γ*) cytokines and increase the levels of Th2-associated cytokines (IL-4, IL-5, and IL-13) in PBMCAs and purified T cells. ATRA promotes the expression of Th2 cytokines rather than Th1 cytokines, as observed in many cell lines [21]. In this study, ATRA-NLCs increased zymosan-induced IL-4 and IL-10 expression and inhibited zymosan-induced IFN-*γ* expression in a dose-dependent manner by cytokine release assay and RT-PCR detection, which means that ATRA-NLCs can decrease fungi-associated corneal inflammation molecular expression.

Zymosan-induced MMP-9 expression at the single-cell level during peritonitis is a quantitative indicator of the phase-specific contribution of mast cells, macrophages, and neutrophils [22]. Zymosan-induced MMP-9 production in neutrophils also affects matrix remodeling [23]. This study investigated the effects of ATRA on zymosan-induced inflammation in corneal fibroblasts, including the regulation of matrix metalloproteinases (MMPs)/tissue inhibitor of metalloproteinase (TIMP). Previous studies have demonstrated that vitamin A and carotenoids regulate immune function in lymphocytes and splenocytes and that the carotenoid lutein regulates matrix metalloproteinase-9 (MMP-9) production in macrophages [24]. ATRA also inhibited TGF-*β*-induced collagen gel contraction mediated by human corneal fibroblasts by attenuating the production of MMP-1 and MMP-3 [25]. ATRA downregulated MMP expression to exert an antimetastatic effect, induce differentiation and apoptosis, and inhibit proliferation and inflammation [24]. ATRA may inhibit MMP expression in different diseases and act as a strong anti-inflammatory agent [18, 24]. The data show that ATRA-NLCs can inhibit the zymosan-induced expression of MMP-1, MMP-3, and MMP-13 and TIMPs in corneal fibroblasts. This effect may be attributed to an imbalance in the expression of proteolytic enzymes and their inhibitors, as described previously [26]. In brief, TIMP-1 and TIMP-2 are produced and related to MMP-1 and MMP-9 in the context of corneal injury. The ratio of MMP/TIMP controls inflammation and is an indicator of tissue damage. ATRA-NLCs can attenuate MMP/TIMP expression and thus could potentially inhibit FK pathology process.

TLR_2_, a regulatory element in the innate immunity of corneal keratocytes, rapidly and transiently induces inflammatory mediators. Antagonists of TLR_2_ may favor the inhibition of signaling responsible for autoimmune responses [27, 28]. Our data show that ATRA-NLCs inhibited the production of inflammatory mediators, that is, MMPs, induced by the TLR_2_ ligand zymosan, and served as an inhibiting feedback regulator of TLR_2_ stimulation.

The pharmacokinetics of potentially therapeutic peptides for the ocular surface, including the residence time of peptides at the cornea, their ability to penetrate the cornea, and the stability of the peptides, has been described [29]. Many studies have attempted to improve the efficacy of therapeutic peptides by improving the residence time. The low bioavailability and poor therapeutic response of traditional ophthalmic formulations is due to a reduced precorneal residence time of the formulation at the ocular surface [30]. NLCs can improve the oral bioavailability of poorly water-soluble drugs. Specifically, NLCs carrying drugs were more effective than the free drugs were because they increased the drug bioavailability [31]. ATRA is highly lipophilic and poorly soluble in water [26], and NLCs were able to prolong the release of ATRA [32]. ATRA-NLCs in this study showed light resistance, proper size, and good physical stability.

The results of this study suggest that ATRA negatively regulates the expression of MMPs, TIMPs, TLR_2_, and IFN-*γ* and positively regulates IL-4 and IL-10 expression. Moreover, ATRA-NLCs play major roles as anti-inflammatory agents in the context of infection via the factors described herein. Thus, ATRA may be useful as an anti-inflammatory agent in the treatment of FK.
